# Probing Catalytic Sites and Adsorbate Spillover on Ultrathin FeO_2–*x*_ Film on Ir(111) during CO Oxidation

**DOI:** 10.1021/acsnano.3c11400

**Published:** 2024-02-20

**Authors:** Hao Yin, Yu-Wei Yan, Wei Fang, Harald Brune

**Affiliations:** † Institute of Physics, École Polytechnique Fédérale de Lausanne (EPFL), 1015 Lausanne, Switzerland; ‡ Department of Chemistry, Collaborative Innovation Center of Chemistry for Energy Materials, Shanghai Key Laboratory of Molecular Catalysis and Innovative Materials, Fudan University, Shanghai 200438, China

**Keywords:** iron oxides, CO oxidation, surface science, hydrogen spillover, model catalyst

## Abstract

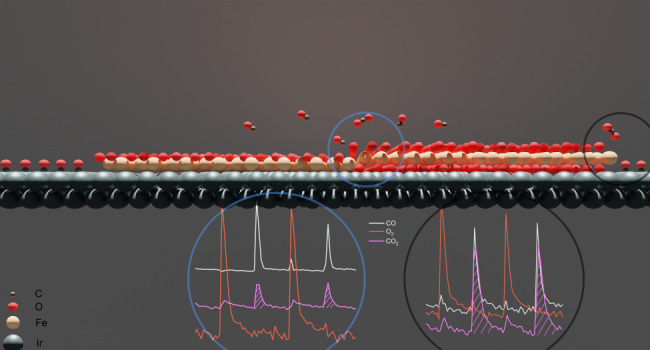

The spatially resolved identification of active sites on the heterogeneous catalyst surface is an essential step toward directly visualizing a catalytic reaction with atomic scale. To date, ferrous centers on platinum group metals have shown promising potential for low-temperature CO catalytic oxidation, but the temporal and spatial distribution of active sites during the reaction and how molecular-scale structures develop at the interface are not fully understood. Here, we studied the catalytic CO oxidation and the effect of co-adsorbed hydrogen on the FeO_2–*x*_/Ir(111) surface. Combining scanning tunneling microscopy (STM), isotope-labeled pulse reaction measurements, and DFT calculations, we identified both FeO_2_/Ir and FeO_2_/FeO sites as active sites with different reactivity. The trilayer O–Fe–O structure with its Moiré pattern can be fully recovered after O_2_ exposure, where molecular O_2_ dissociates at the FeO/Ir interface. Additionally, as a competitor, dissociated hydrogen migrates onto the oxide film with the formation of surface hydroxyl and water clusters down to 150 K.

## Introduction

Well-defined ultrathin, down to one atomic monolayer thick, oxide films on single-crystal metal surfaces are widely recognized as inverse metal-oxide model catalysts^1,2^ that are ideally suited to disentangle the structure-performance relationship and to elucidate key catalytic concepts such as the identification of active sites, the spatiotemporal distribution of surface species, strong metal–support interaction, and more. Compared with bulk materials, two-dimensional oxide films can possess unique stoichiometries and electronic properties resulting from substrate interaction, as well as from spatial confinement. Among varieties of systems, reducible transition metal oxides (TMO) (e.g., FeO_*x*_, CuO_*x*_, CoO_*x*_) draw great attention, especially toward oxidation reactions such as low-temperature CO oxidation, water–gas shift reaction, and preferential oxidation, typically involving lattice oxygen in the reaction. One representative ultrathin TMO system is a monolayer FeO_*x*_ film grown on platinum-group metals (PGMs) (Pt,^3,4^ Pd,^5^ Ru,^6^ Ir^13^), where the local structure reversibly transforms between O-rich (O–Fe–O trilayer) and O-poor (O–Fe bilayer), depending on the chemical environment. The charge transfer at the oxide/metal interface stabilizes the polar FeO_*x*_ surface in ultrahigh vacuum (UHV) conditions and weakens the Fe–O bond in the topmost layer of the FeO_2_ trilayer. The weakly bound oxygen (WBO) atoms potentially facilitate CO oxidation via the Mars–van Krevelen (MvK) mechanism.^7^

For FeO_*x*_/PGMs systems, many works in UHV conditions focused on the identification of surface species and of the active phase. For example, Bao^8^ and co-workers concluded that the interfacial FeO/Pt sites are active for dioxygen dissociation producing individual reactive oxygen atoms and promoting selective CO oxidation in the Pt–Fe/SiO_2_ powder system. Wendt et al.^9^ directly visualized the O-edge of FeO as active sites. At a higher oxygen partial pressure, a bilayer FeO/PGMs surface is gradually oxidized into a trilayer FeO_2–*x*_/PGMs surface. Pan et al.^10^ inferred that the reaction occurs at the FeO_2_/Pt interfaces, which have a similar structure than the FeO/Pt interface. Only recently has the interface between O-rich/O-poor domains gathered more attention as alternative active site. Zhang et al.^11^ pointed out that CO oxidation takes place at the FeO_2_/FeO interface, regardless of film coverage in clear contrast with the conclusions drawn in refs (9) and (10). Therefore, it is not entirely clear which role these different sites really play under which reaction conditions. This can be addressed by isotope-labeled pulse reactions with real-time product detection, which, to the best of our knowledge has not been done so far. Another aspect that has not been addressed is how the reaction pathway is changed in a more reducing environment, such as preferential oxidation conditions with the coexistence of hydrogen, the participation of hydrogen and surface water.^12^

In this work we combine variable-temperature scanning tunneling microscopy (VT-STM) with a very sensitive home-built chemical analyzer to study the CO oxidation on ultrathin FeO_*x*_ films grown on Ir(111). Real-time isotope-labeled reaction product analysis and atomic scale surface morphology imaging indicate two distinct active reaction sites. At 500 K, we observe initially a rapid CO_2_ production where CO reacts with WBO at the FeO_2_/Ir interline. Afterward, the CO oxidation continued at the FeO_2_/FeO interface, yet with a more than 1 order of magnitude lower reaction rate until the surface turned completely to FeO. The trilayer FeO_2_ structure can be regenerated after O_2_ exposures at 570 K. Motivated by the role played by preferential oxidation in industrial CO oxidation, we also investigated the interaction of FeO_*x*_/Ir with hydrogen. Real-space images demonstrate that hydrogen atoms directly consume the weakly bonded oxygen at the metal/oxide interface with a disappearance of the FeO_2_/Ir interfacial WBO trilayer and a generation of surface hydroxyls and water clusters. High-temperature O_2_ exposures could reactivate the hydroxylated surface to a large extent with a very low density of hydroxyls/water species remaining. Surface hydrogen spillover exists within a large temperature window and strongly contributes to the consumption of the active oxygen phase and thereby competes with CO oxidation.

## Results

### O-Rich/O-Poor Structures of FeO_*x*_ on Ir(111)

**Figure 1:**
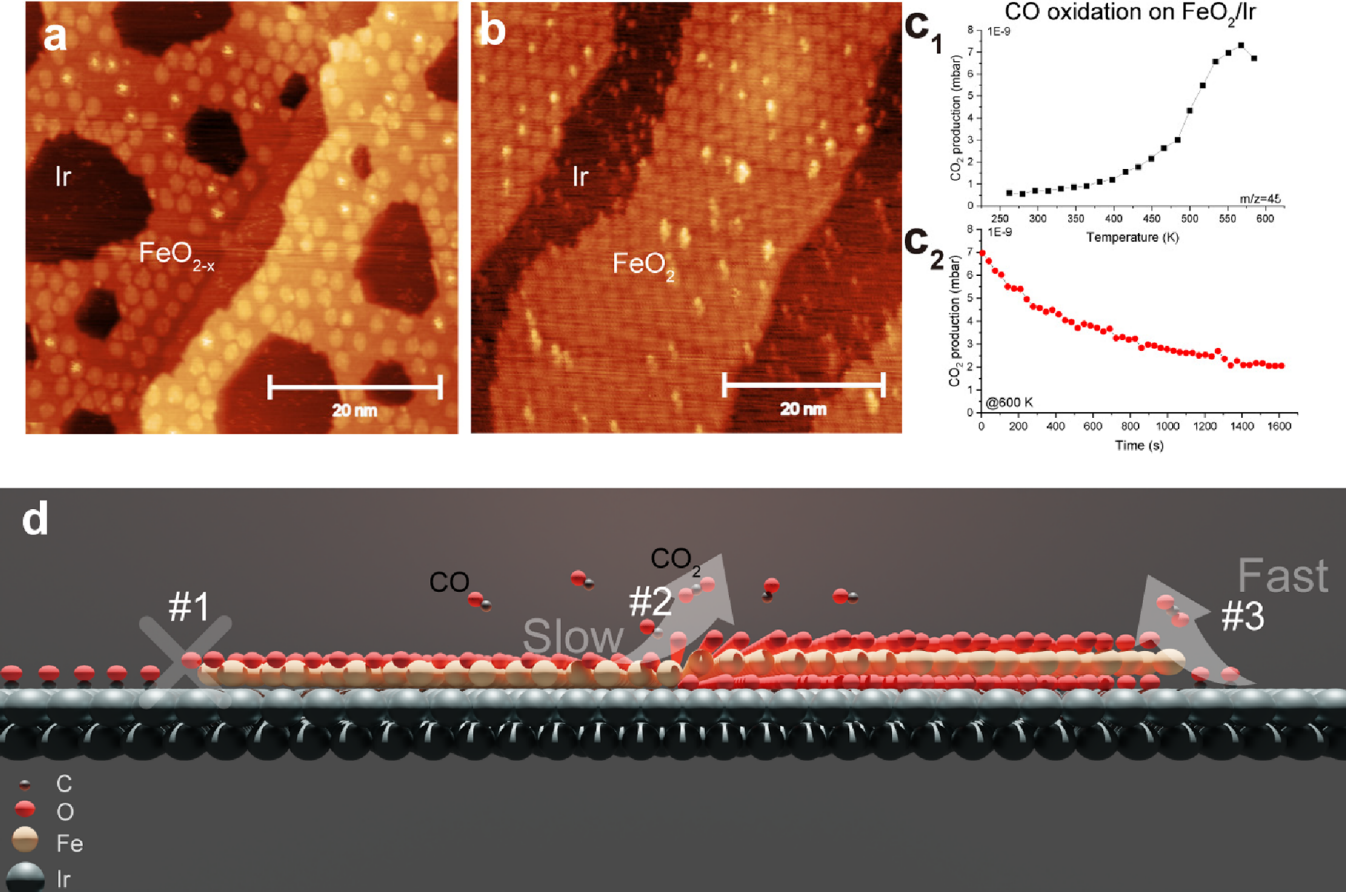
(a, b) STM images (*V*_t_ = 2 V, *I*_t_ = 0.1 nA) of 0.6 ML FeO*_x_*/Ir(111) coexisting with clean Ir(111) (300 K, 1.3 × 10^–7^ mbar O_2_) after different thermal and oxygen exposures: (a) 700 K, 1 × 10^–6^ mbar O_2_ 120 s; (b) 700 K, 1 × 10^–6^ mbar O_2_ 300 s and 570 K, 5 × 10^–6^ mbar O_2_ 120 s. (c_1_) Temperature-dependent CO oxidation performance (CO and O_2_ pulse cycle, each pulse with 1 × 10^–6^ mar, 0.2 s duration); (c_2_) Time-dependent CO oxidation at 600 K (CO dose only) for FeO_2_/Ir(111). (d) Art scheme of active sites on FeO_2–*x*_/Ir during CO oxidation: FeO_2_/FeO and FeO_2_/Ir interfaces are active, whereas the FeO/Ir interface is inactive.

A sample with a partial FeO coverage of 0.6 monolayer (ML) on Ir(111) was prepared by Fe evaporation in an oxygen background pressure and subsequent annealing in oxygen (for parameters see Methods). Typically, the PGMs-supported bilayer FeO films are trigonal structures where the Fe layer is located between the terminating O layer and the PGMs substrate. Due to a lattice mismatch between FeO and Ir(111), similar to the FeO/Pt(111) system,^14^ a moiré pattern is formed.^13^ After annealing in an oxidizing environment (5 min, 700 K, 1 × 10^–6^ mbar O_2_), the bilayer partially transforms into a trilayer structures. Figure 1a shows an STM image revealing three apparent heights, the Ir(111) surface, the FeO layer, and protruding from it by 0.5 Å small round FeO_2_ domains. These domains show a preferred distance of about 20 Å^13^ (Figure S1). The topmost oxygen layer in both structures is weakly bound (WBO) to the Fe layer, as evidenced by a low-temperature oxygen desorption peak in temperature-programmed spectroscopy (TPS). Once we further expose this FeO_*x*_ surface to oxygen (2 min at 570 K, 5 × 10^–6^ mbar O_2_), the surface almost fully transforms into the FeO_2_ trilayer that exhibits a periodic pattern, as seen in Figure 1b. Similar to Sun et al.,^14^ we also notice that some higher protrusions appear on the FeO_*x*_ superstructure without a certain regular pattern, which are likely hydroxyl species^15^ or water.^16−18^

### Isotopically Labeled CO Oxidation on FeO_*x*_/Ir(111)

We measure CO oxidation and TPS by a home-built very sensitive chemical analyzer^19,20^ with millisecond time resolution and a detection limit of 10^–10^ mbar (CO_2_). It allows us to dose and analyze gas molecules on the surface in a separately pumped volume with different pressures (up to ∼10^–5^ mbar) without significant change of the pressure in the main STM chamber (maintaining ∼10^–10^ mbar). This allows us to take out all measurements in a single UHV chamber. Figure 1c_1_ displays the CO_2_ production from FeO_2_/Ir(111) measured by alternatingly pulsing ^13^CO and O_2_ (each pulse interval ∼12 s) while annealing the sample from 250 to 600 K. We use isotopically labeled ^13^CO to selectively detect reactant molecules and discern them from ^12^CO in the residual gas. The displayed CO_2_ production is synchronized with the CO pulses (labeled as CO_2_(CO)). The one synchronized with the O_2_ pulses (labeled as CO_2_(O_2_)) is very small and stems from the Ir(111) surface (Figure S4). Similar to Pt(111), at room temperature, the reaction on Ir(111) is limited by the small number of available sites for the O_2_ dissociation due to the strongly bound CO molecules. With increasing temperature, the CO_2_ production rate increases up to 550 K, from where it decreases due to CO desorption (Figures S3 and S4). Due to a strong interaction between CO and PGMs, CO molecules prefer adsorbing on Ir(111) instead of oxide films. Therefore, we estimate main reactive sites are likely located at the boundary between FeO_2_ and Ir where adsorbed CO molecules react with WBO following the MvK mechanism up to 550 K, as schematically shown in Figure 1d in process #3. In Figure 1c_2_, we investigate the reaction dynamics only with CO doses at 600 K on FeO_2_/Ir(111). The reaction rate decreases by more than 60% within 20 min due to the consumption of FeO_2_/Ir(111) interfacial WBO. After that, the reaction continues with a lower rate at the FeO_2_/FeO boundary, see Figure 1d process #2 until the trilayer O–Fe–O structures fully transform into bilayer Fe–O structures.

**Figure 2:**
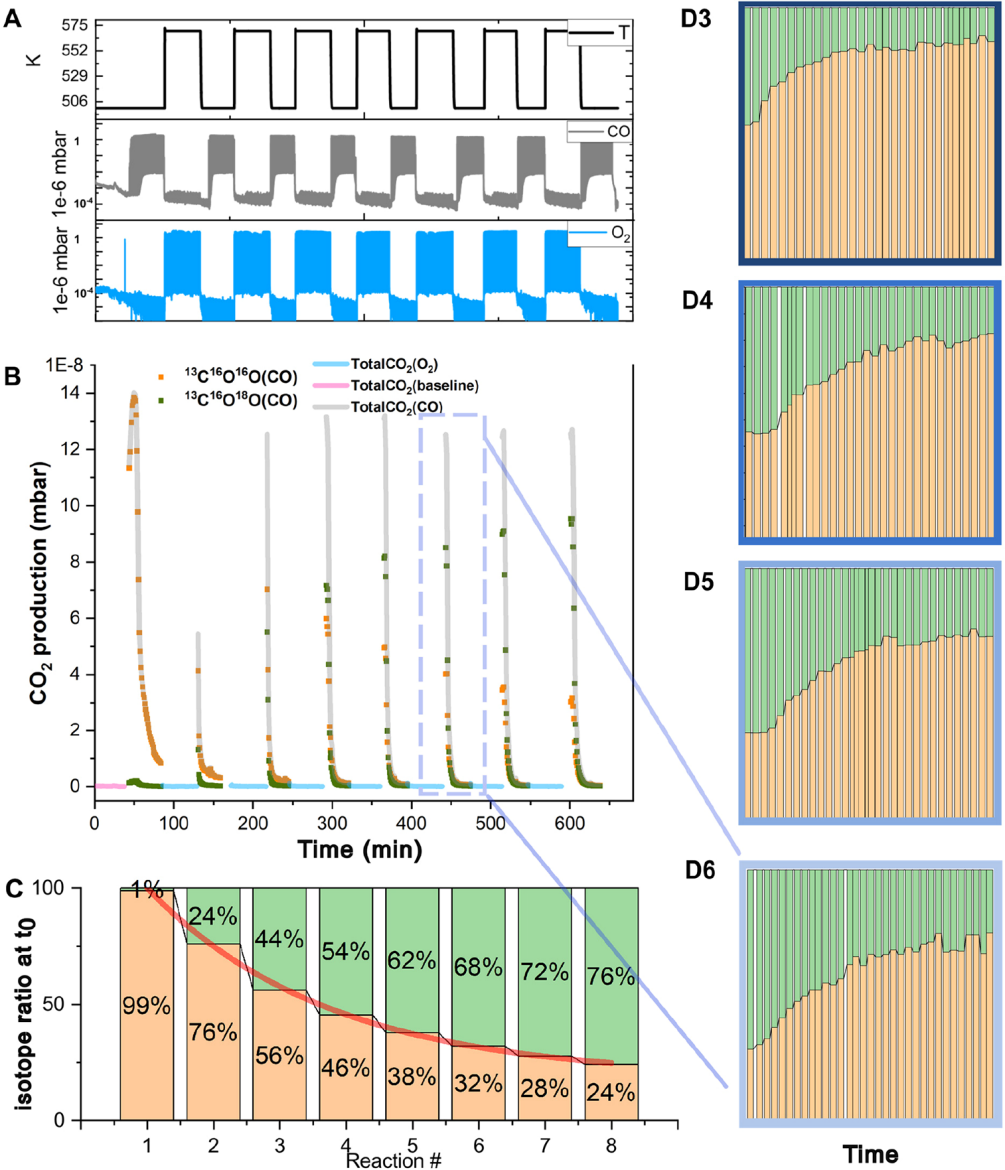
(A) CO oxidation conditions: sample temperature, CO dose, and O_2_ dose. (B) CO_2_ production cycles after CO and O_2_ exposures repeatedly. Reaction activities dramatically drop within 30 min. Meanwhile, after O_2_ exposures, the reactivity recovered to the initial level. (A) and (B) share the same *x*-axis. (C) The initial *t*_0_ CO_2_ isotopic ratio (^13^C^16^O^18^O: green, ^13^C^16^O^16^O: brown) develops with reaction cycles where FeO and CO are the sources of ^16^O and O_2_ is the source of ^18^O. (D) Isotope-ratio changes in certain continuous CO pulse periods within 30 min. (D3)–(D6) refer to the reaction # shown in (C).

**Figure 3:**
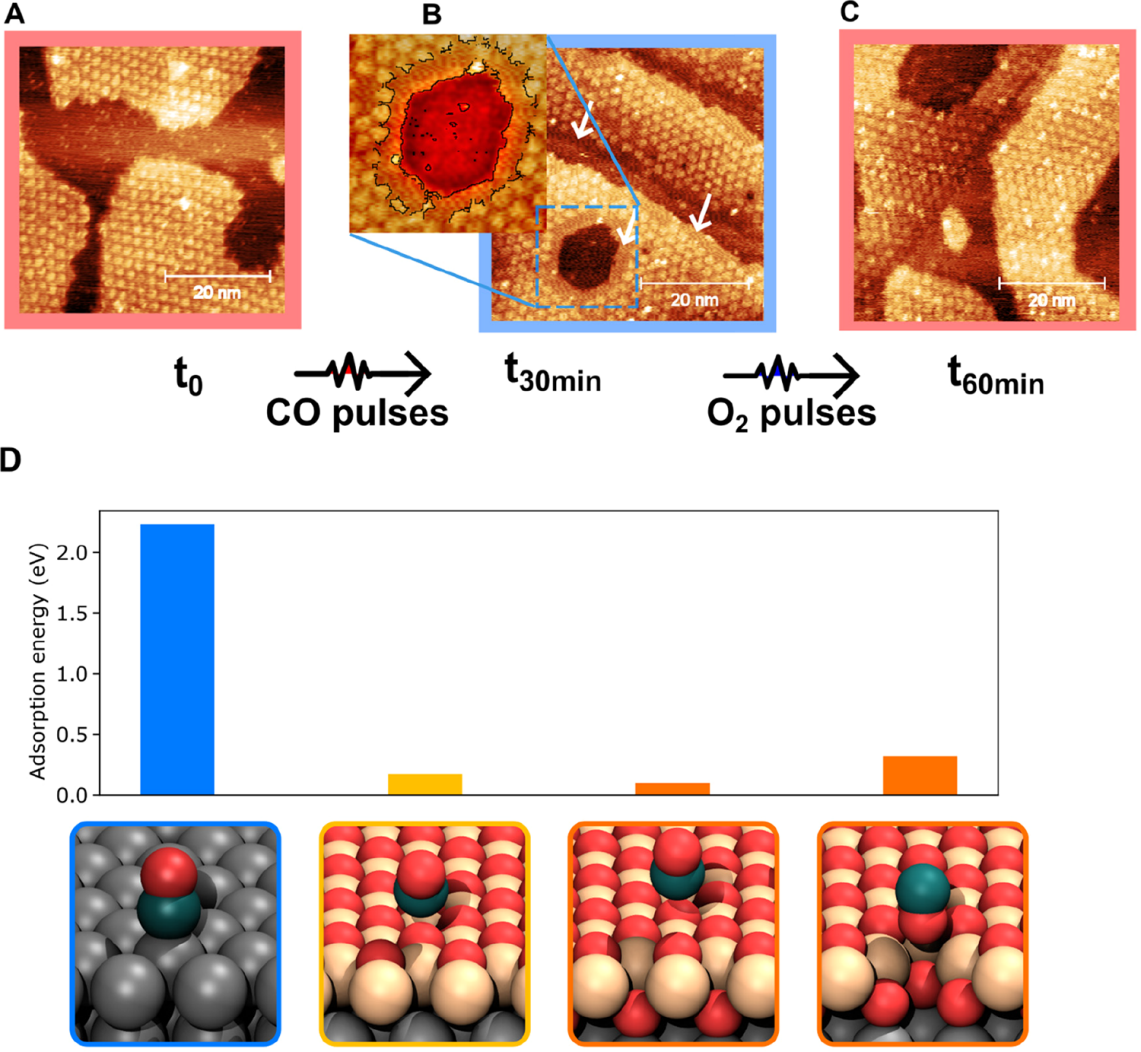
STM images of the FeO_2_/Ir(111) surface at different reaction stages. (A) Initial state before reaction; the surface consists of FeO_2_ islands that exhibit a moiré pattern and the bare Ir(111) surface. (B) After CO pulses for 30 min, one sees that the FeO_2_ island edges have transformed into a FeO bilayer that is imaged flat (see arrows), a blue dotted box highlights a flat “rim” with outline; (C) After ^18^O_2_ exposure for 30 min, the FeO_2_ islands are fully regenerated. (D) From left to right: CO adsorption energy on Ir, FeO, FeO_2_, and O-missing defect sites. The atoms are colored as follows: C, dark blue; Fe, yellow; O, red; Ir, gray.

To determine the dominant oxygen species involved in the reaction, we trace the dynamics of CO_2_ production on FeO_2_/Ir with isotope-labeled reactants (^13^C^16^O, ^18^O_2_, and Fe^16^O_2_). Different from the parameters in Figure 1c_1_, we prolong the duration of the CO and O_2_ dosage to 30 min to fully separate the reduction environment from the oxidation one. We alternate the sample temperature between 500 K during the CO dose and 570 K during the O_2_ dose at which temperature most CO on Ir is almost desorbed. Since we operate the valves not in their foreseen fully open mode, there are certain fluctuations in gas doses, especially for the O_2_ dose. Nevertheless, the total O_2_ dose each cycle is >50 Langmuir (L) enabling most surface FeO to be fully oxidized after each O_2_ cycle. The time-dependent CO_2_ production during eight CO-O_2_ cycles are displayed in Figure 2B, and the corresponding fractions of CO_2_ isotopes at the initial pulse (^13^C^16^O^16^O marked in brown vs ^13^C^16^O^18^O marked in green) in Figure 2C. In this condition, the interfacial trilayer films transform into the bilayer FeO films as far as 3 nm from the FeO_2_/Ir interline. See in Figure 3B the 3 nm wide flat rim surrounding a 10 nm diameter hole in the FeO_*x*_ and a similar rim initiating from the Ir step edge. All three locations are marked by white arrows. Even though a large portion of the remaining “less active” FeO_2_ superstructures still exist on the surface at *t*_30min_, the reaction performance from that moment is not comparable to the one at the initial *t*_0_ state for each cycle. The weakly bonded oxygen trilayer structures at the FeO_*x*_/Ir interfaces can be regenerated after O_2_ exposures at 570 K, as described in Figure 3C, with the recovery of total reactivity in Figure 2B.

Another strong evidence to support the MvK mechanism is the fact that the dominant product isotopes shift from ^13^C^16^O^16^O to ^13^C^16^O^18^O at the peak reaction rate as described in Figure 2C. As mentioned, interfacial trilayer weakly bonded oxygen is constantly consumed at the CO reducing period and regenerated at the ^18^O_2_ oxidizing period. Consequently, the topmost layer of oxygen atoms at the FeO_2_/Ir interface is gradually replaced from the initial ^16^O to ^18^O, which further directly participates in ^13^C^16^O oxidation. As shown in Figure S8, once we increase the ^18^O_2_ exposure from 50 L to >200 L in each oxidation period, the percentage of ^13^C^16^O^18^O in the second cycle rapidly increases to 58% compared with 24% in Figure 2C. The isotope exchange rate of ^16^O against ^18^O atoms is positively correlated with O_2_ pressure, which indicates that the rate-limiting step for WBO regeneration is most likely the dissociation of oxygen on the surface. Similar to former reports on FeO film on Pt(111),^8^ we estimate that molecular oxygen likely dissociates at the boundary of FeO and Ir(111). Surprisingly, a reverse isotope-portion trend in a single cycle is observed (Figure 2D3–D6) where the proportion of ^13^C^16^O^16^O gradually increases with a decline in reaction rate. A rational explanation would be that the noninterfacial Fe^16^O_2_ which remains unaffected during O_2_ pulse exposures also contributes to CO_2_ production with a much lower frequency. We also observe a larger depletion of trilayer WBO after 60 min CO exposures from the STM image (Figure S7) compared with the one after 30 min in Figure 3B. Coincidentally, Zhang et al.^11,21^ recently emphasized that CO oxidation can take place at the interface between FeO_2_ and FeO on Pt(111) by *in situ* STM measurement. Based on the performance results and the STM image, we interpret that the CO_2_ molecules at the initial moment *t*_0_ generated from the FeO_2_/Ir interface (MvK mechanism) and the one at 30 min moment *t*_30min_ likely originate from the FeO_2_/FeO interface. Temperature-dependent measurements in Figure S6 reveal the apparent activation energy (*E*_a_), which (35.78 kJ/mol for *t*_0_) is relatively similar to Pt/FeO_*x*_ catalysts reported from other groups,^21,22^ indicating similar rate-limiting steps.

We noticed that in Figure 2B the CO_2_ production in each reaction cycle presents a short increasing stage in the beginning. This phenomenon may result from CO adsorption behaviors on Ir(111). Initially, compared with free CO molecules in the gas phase, insufficient surface CO (∼0.2 L per pulse) molecule coverage partially limits the reactivity at the FeO_2_/Ir interface, indicating that the rapid reaction stage starts with CO surface adsorption. With an increase in surface CO coverage, the performance gradually reaches the maximum and the reaction rate is limited from there on by interfacial WBO consumption. We also measure the alternate CO/O_2_ pulse-related catalytic oxidation performance with shorter time intervals (seconds) on FeO_2–*x*_/Ir(111) systems (Figure 1a) with fewer interfacial trilayer coverage at 500 K. Compared with FeO_2_/Ir surface (Figure 1b), it is noteworthy that there is a low-reactivity phase before the normal rapid oxidation (Figure S5). This phase is attributed to the lack of interfacial WBO at the earlier stage which may result from insufficient oxidation during sample preparation (oxygen vacancy), and the CO oxidation rate at the FeO_2_/FeO interface is much slower. Once the bilayer structures turn to the trilayer active phase at the FeO_2_/Ir interface, the rapid reaction stage becomes renascent. These findings suggest FeO_2_/Ir interfacial WBO species are recognized as the active phase during rapid catalytic CO oxidation with the MvK mechanism, similar to former research on the FeO_*x*_/Pt(111) surface.

DFT calculations were conducted to obtain insight into the reactivity of CO with WBO and its active sites. First, we examined the adsorption sites for CO on the surface. Adsorption geometries were optimized on Ir, FeO, and FeO_2_ terraces, and adsorption energies are compared in Figure 3D. The adsorption energy is defined as *E*_ad(X)_ = −(*E*_total(X)_ – *E*_surf_ – *E*_*x*_), where *E*_total(X)_ is the total energy of whole system (X adsorbed on the surface), *E*_surf_ is the energy of the relaxed surface, and for CO adsorption, *E*_*x*_ is the energy of a gas-phase CO molecule. One can see that CO is chemisorbed on Ir(111) with a strong adsorption energy of 2.1 eV, while it can only physisorb on FeO or FeO_2_ via weak van der Waals interactions. With rather low effective collision frequency due to weak adsorption, at our given condition, gas-phase CO molecules hardly directly react with terrace oxygen at the FeO_2_/Ir film. Energy differences (Δ*E*) between the reactant and an intermediate state (modeled in a 4 × 4 cell) are estimated 1.8 eV (Figure S10). This finding shows that #3 in Figure 1d is the preferred reaction site as it can bind CO to the surface, enabling the MvK mechanism, and is consistent with previous studies.^8^

Qualitatively, we estimate that oxygen atoms at FeO_2_/FeO edge sites are weakly bonded and more reactive compared to terrace sites, as they have a lower coordination number with Fe atoms. This observation explains why the further depletion of WBO primarily occurs at the FeO/FeO_2_ boundary rather than being randomly distributed throughout the terrace of the FeO_2_ film. Additionally, we also observe some reactive sites on the terrace, as indicated by the circles in Figure S7. Those active sites may originate from local oxygen vacancies. In Figure 3D, it is evident that oxygen vacancies on FeO_2_ have a greater affinity for CO molecules compared to the terrace sites. This increased affinity is indicated by a 0.2 eV higher CO adsorption energy. In other reports on FeO_2_/Pt(111), it appears that smaller FeO_2_ islands (20 nm diameter) at lower Fe coverage have lower oxygen vacancy concentrations.^21^ We attribute the existence of oxygen vacancies to heterogeneity during larger film growth, consistent with the aforementioned details in Figure S5.

**Figure 4:**
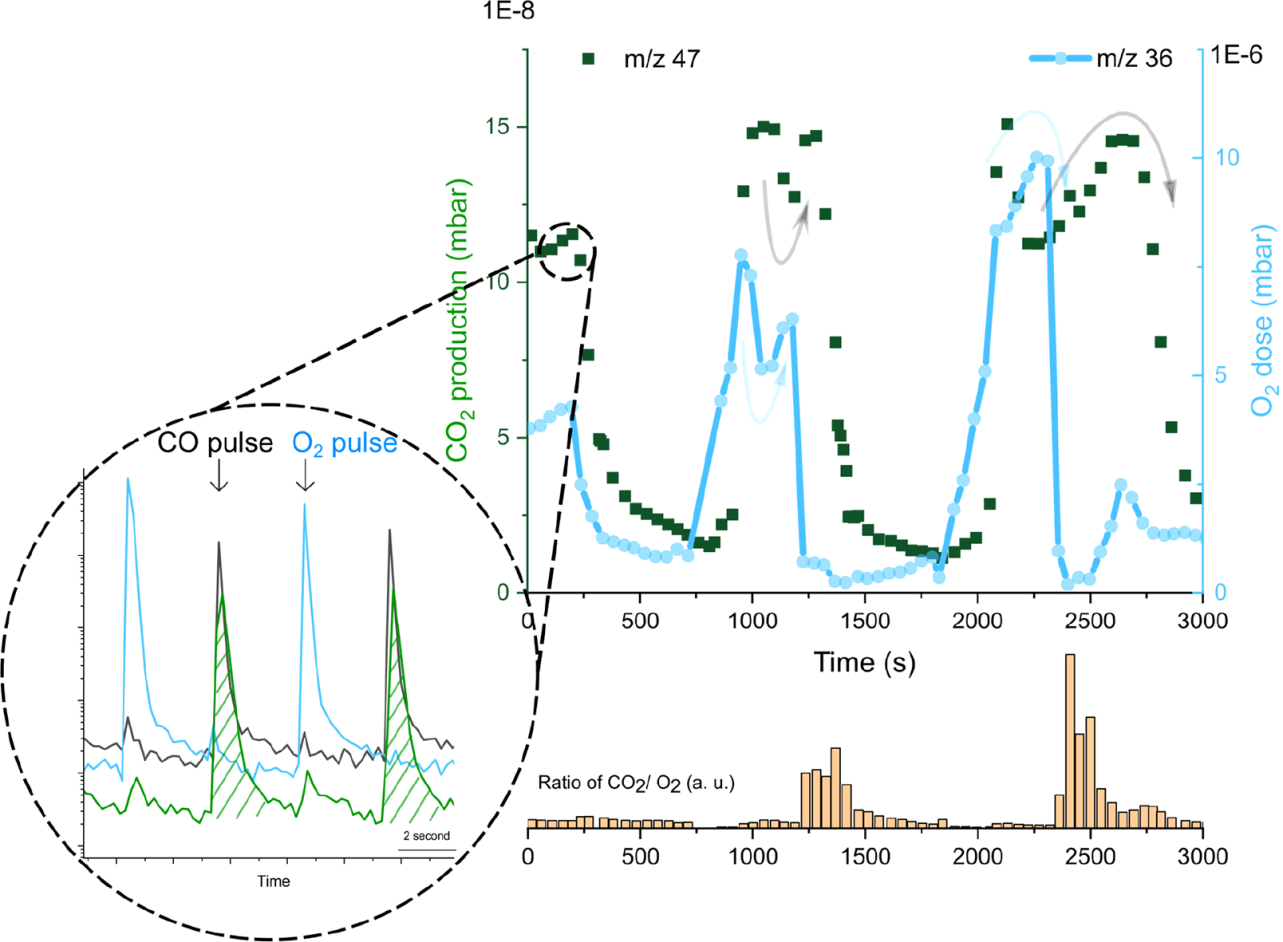
Inside circle: alternating pulses of O_2_ (blue), CO (gray), and CO_2_(CO) (green) as shown as a function of time. Main: the integrals of the O_2_ pulses (blue) and CO_2_ peaks synchronized with CO pulses (green dots) are reported versus the time at 500 K. Bottom: intensity ratio of CO_2_(CO) and O_2_, clear delays were observed at ∼1300 s and ∼2500 s where CO_2_(CO) remains with a decreasing O_2_ supply.

To further describe the participation of oxygen, compared with a constant amount of CO pulse dose, we adjust the amount of O_2_ pulse dose during the pulse experiments with short time intervals at 500 K. Each cycle consists of one CO pulse and one O_2_ pulse (Figure 4, left). We focus on the amount of CO_2_ synchronized with CO pulses (CO_2_(CO)) where trace CO_2_ during the O_2_ pulses (Figure 2B) is possibly generated from the bare Ir(111) surface (Figure S4). As shown in Figure 4, we observed a positive correlation between the amount of O_2_ and CO_2_(CO) generation under the same CO pulse pressure, which is similar to Zhang et al. results.^11^ Within a threshold, the more oxygen we dosed, the faster the active phase is regenerated. Once we dosed oxygen above 1.2 Langmuir per pulse (CO/O_2_ = 1:5), the interfacial WBOs recovered much faster than they consumed during the CO pulse, and CO_2_ production reached a plateau irrelevant to the extra amount of O_2_. We contribute this phenomenon to a limit of the O_2_ kinetics dissociation once O_2_ adsorption/desorption reaches equilibrium with all surface sites. Interestingly, there seems to be a time delay for CO_2_ responding to O_2_ changes. It also indicated that molecular oxygen does not directly participate in CO oxidation but shuffles the oxidation state of FeO.

### Hydrogen as a Competitor

FeO_*x*_/PGMs catalysts potentially promote low-temperature CO oxidation. In addition, they possess great potential in preferential oxidation (PROX) reactions.^33^ This is important as there is as a demand from the hydrogen purification industry to remove trace amounts of CO from H_2_/CO mixtures. Therefore, it is essential to reveal the competing role of H_2_ and the mechanism behind it. In addition, thin oxide films on metals are ideal model systems to investigate PROX.^23^

**Figure 5:**
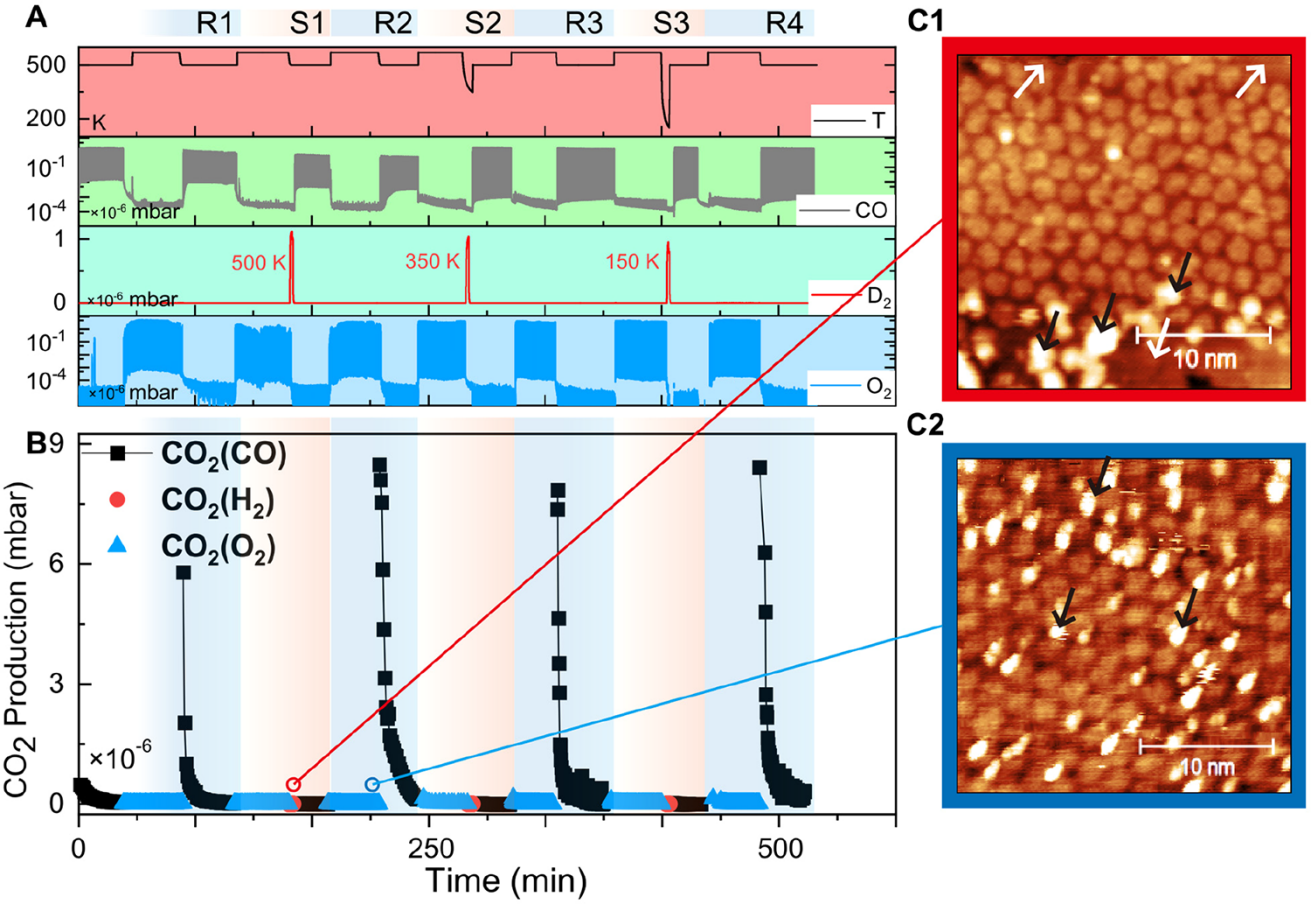
(A) CO oxidation conditions with deuterium coadsorption: Temperature (black), D_2_ dose (red), CO dose (gray, log scale), and O_2_ dose (blue, log scale). (B) CO_2_ production cycles after CO and O_2_ with/without hydrogen coadsorption repeatedly. We dose D_2_ for 2 min at 500, 350, and 150 K, where the CO reactions were all impeded after H_2_ exposures. (C1, C2) STM images of FeO_2_/Ir at different reaction stages. (C1) After ^18^O_2_ + D_2_ exposures; (C2) The hydrogenated surface after ^18^O_2_ exposures. After hydrogen exposures (C1), similar to CO exposures, the interfacial O–Fe–O trilayer disappeared (white mark), while larger protrusions appeared (black mark).

**Figure 6:**
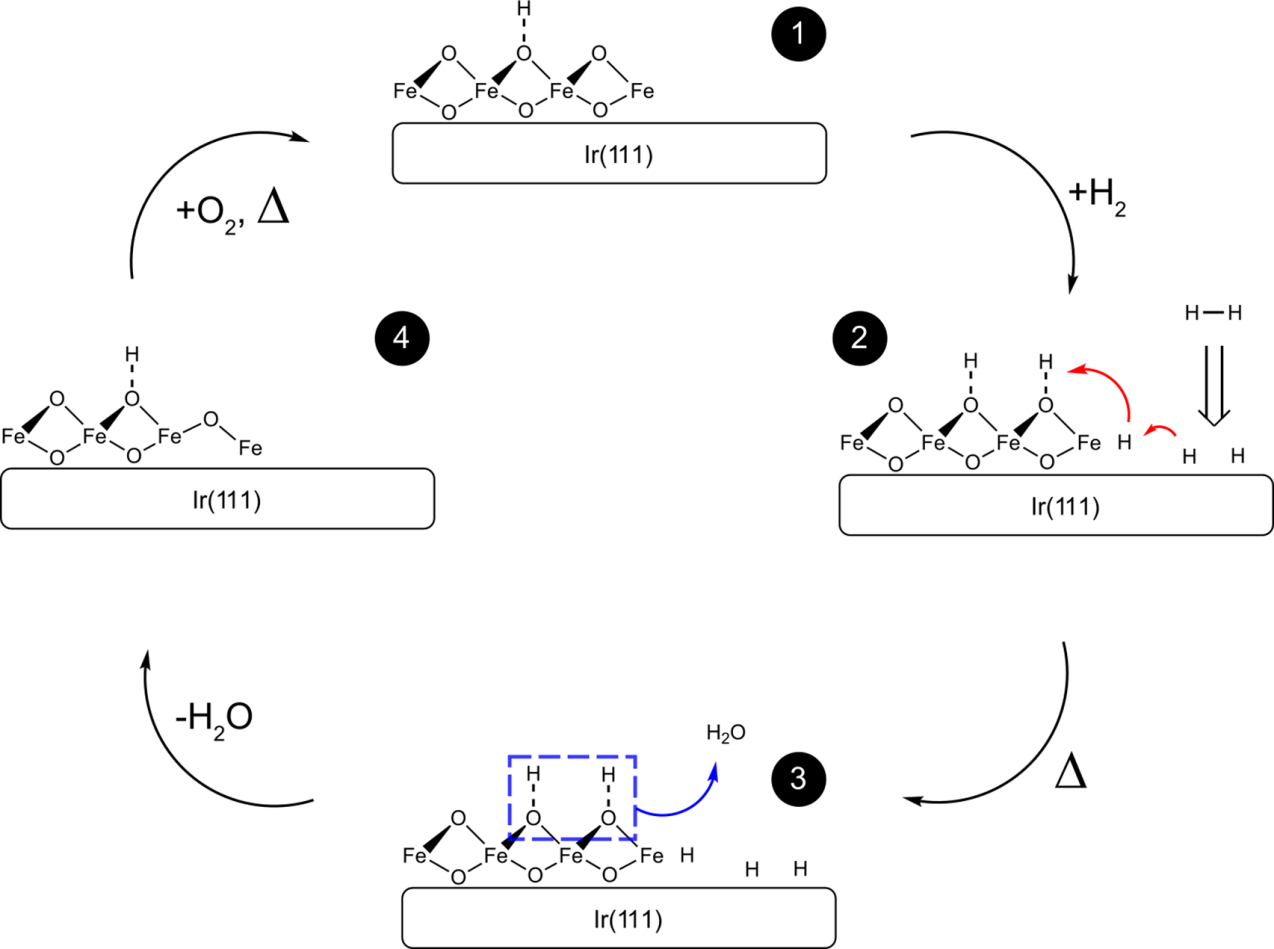
Brief schematic of hydrogen coadsorption and spillover process on FeO_2_/Ir(111) surface.

We dose approximately 100 L deuterium (D_2_) via a leak valve after the oxidizing period at three temperatures: 500, 350, and 150 K, see Figure 5A. The total O_2_ exposures during each interval are between 80 and 100 L which is sufficient for the full reoxidation of the sample. Even though the bilayer FeO_2–*x*_/Ir sample (Figure 1) is weakly reactive in the first CO period, interfacial WBO species are partially regenerated after O_2_ exposures, as well as with a recovery of CO oxidation performance in the R1 cycle (Figure 5B). Interestingly, the reactivity seems to largely decline after hydrogen exposures both in cycles S1, S2, and S3 at all three H_2_ exposure temperatures. Surface characterization reveals a similar morphology as dedicated CO exposures in Figure 5B where the topmost oxygen atoms on the FeO_2_/Ir interface are consumed (white mark in Figure 5C1) after H_2_ exposures at 500 K, yet a major portion of trilayer structures far from the boundary remain undisturbed. There are also >5 Å protrusions (black mark in Figure 5C1, height profile seen in Figure S9) forming around the trilayer/bilayer rim compared with the situation during CO exposures. Other groups observed similar protrusions on both FeO/Pt film^15,16^ and bulk Fe_3_O_4_(001) surface,^17,24^ where these protrusions are identified as water agglomerates or hydroxyl species depending on different conditions. Due to the strong H_2_-affinity of PGMs, hydrogen molecules are known to dissociatively chemisorb on Ir(111) at even close to liquid nitrogen temperature (90 K).^25^ Hydrogen atoms from the metallic catalyst surface can further migrate onto the support oxides, also known as hydrogen spillover. The atomic hydrogen mobility on oxides is normally several orders of magnitude lower than that on PGMs, which restricts the spillover effect to nanoscale distances from the metal-oxide boundaries. Still, similar to the reported PGMs on iron oxides system,^28^ we also attribute the active region to the fact that the initial OH species generated at the Ir/FeO_2_ boundary do not significantly impede the subsequent spillover of hydrogen atoms compared with nonreducible oxides such as γ-alumina(100).^29^ The proposed H_2_ reaction scenario is described in Figure 6. We interpret the hydrogen oxidation with the successive process where the hydrogen dissociatively adsorbs on Ir(111) and migrates onto FeO_2_ films at down to 150 K with the formation of oxyhydroxide species, and the topmost WBOs were consumed by further dissociation of surface oxyhydroxide species and desorption of water clusters at 500 K. Combined with D_2_O temperature dependent desorption results in Figure S11, we contribute the interfacial WBO depletion to the hydroxylation, water formation and desorption above 400 K. A larger reactive region (trilayer WBO depletion) within 10 nm from the boundary is possibly determined by the surface hydrogen spillover process^26,27^ as shown in Figure 5C1. Afterward, CO oxidation is strongly reduced resulting from the lack of active oxygen species, and surface transition from Ir/FeO_2_ interface into FeO_2_/FeO and Ir/FeO interface, which is strongly consistent with the aforementioned CO results.

Similar to the results in Figure 3, the interfacial trilayer structures can be restored after oxidation at 570 K also for the H_2_-treated sample, as shown in Figure 5C2. In addition, a small coverage of oxyhydroxide/water clusters may remain on the surface. At the same time, we observe an escalation in reaction performance during the R2, R3, and R4 CO dose period in Figure 5B. The coexistence of surface oxyhydroxide/water clusters with the active trilayer phase seems neither promote nor impede CO_2_ production. We notice that some previous literature reports considered surface water/oxyhydroxide as a promoter for CO oxidation on metal-oxide systems,^12,30^ while some did not.^31,32^ Further research with advanced spectroscopy methods could be undertaken to investigate specifically the Fe–OH_*x*_/CO interaction on the FeO_*x*_/Ir(111) surface. Our results reveal that surface hydrogen atoms are highly mobile and compete with CO oxidation. They consume interfacial active oxygen by spillover. Hydrogen spillover onto the oxide film can be a rather complicated process, which may strongly be affected by the coordination numbers of surface O sites and local surface geometries, as mentioned by Liu et al.^23^ We find that even though a large number of remaining WBOs on the terrace can participate in further CO oxidation, the reactivity significantly declines.

## Conclusion

Our experimental (STM, TPD, and transient pulse methods) and theoretical (DFT) studies of the CO oxidation on FeO_2_ and on FeO monolayers grown on Ir(111) reveal two active sites. The reaction takes place with weakly bound oxygen in the topmost layer of both surface oxides. It occurs most efficiently at the FeO_2_/Ir(111) interface, followed by the FeO_2_/FeO interface. Co-adsorption of H_2_ leads to a competing reaction. Already at 150 K, hydrogen dissociates and the hydrogen adatoms compete with adsorbed CO molecules for active oxygen species, they migrate onto surrounding FeO_2_ and form oxyhydroxide species. The interfacial depletion region during hydrogen exposure is determined by hydrogen spillover and thermal oxyhydroxide/water cleavage. Both are recognized as competing processes during preferential CO oxidation. In summary, we identify the active sites and adsorbate spillover and thereby provide an improved fundamental understanding of the CO oxidation mechanism, especially when competing with hydrogen.

## Methods

### Experimental Details

Experiments were carried out in a UHV VT-STM chamber (home-built “Beetle”-type STM) with a base pressure below 3 × 10^–10^ mbar, equipped with a QMS, an e-beam evaporator (triple evaporator EFM 3T, Focus), an ion sputter gun (SPECS IQE 12/38), and a multiple gas doser system for oxygen and CO pulses (3.7, PanGas) directed onto the sample surface. A sample manipulator allows for sample heating by radiation and electron-bombardment, as well as for sample cooling with a flux cryostat down to 40 K by LHe, and to 130 K by LN_2_.^20,33^ The temperature is measured by a Ni-Cr/Ni-Al thermocouple (Type K) whose wires are spot-welded onto the brim of the crystal. The reference thermocouple junction is placed in a thermally regulated preamplifier (H. Schlichting, Pureions). The power supplies of the filament are PID-controlled (Eurotherm). The Ir(111) sample (Mateck) was cleaned by repeated cycles of annealing to 1500 K (1 min) and sputtering with a beam of 1.5 keV 3.1 μA/cm^2^ Ar^+^ ions at 300 K. Its cleanliness, as well as the density of structural defects, were controlled using STM. We calibrated the metal deposition rates by determining the fractional coverage from STM images recorded after sub-monolayer disposition of elements for a given time onto the clean Ir(111) surface. This was done under conditions where large islands form, minimizing uncertainties due to tip-convolution. We define the coverage of one monolayer (ML) as one metal atom per Ir(111) surface atom.

For the preparation of FeO_*x*_ on Ir(111), we departed from the parameters used for FeO_*x*_/Pt(111). To form FeO_*x*_ on pristine Ir(111), 0.6 ML Fe (99.99%, Goodfellow) was deposited at room temperature at 1.3 × 10^–7^ mbar O_2_ partial pressure and subsequently annealed for 2–10 min in 1 × 10^–6^ mbar O_2_ to 700 K to reduce the island and form larger terraces. Note that a longer annealing time may increase the coverage of trilayer WBO on FeO_*x*_ film. To prepare the nearly fully trilayer FeO_2_ film, we further annealed the FeO sample in 5 × 10^–6^ mbar O_2_ for 2 min to 570 K. All STM measurements were performed at room temperature.

The reactant gas dosing and reaction product detection system (Sniffer) was built by equipping a commercial QMS (QMA 200, Pfeiffer Vacuum) with a modified ionizer and a detection volume directly connected to the sample surface. The reactant gas pulses are generated with electro-valves (Parker, series 99) operated by a rectangular voltage pulse of typically 1–4 μs duration and 20–30 V amplitude, corresponding to a partial opening of the valve.^19,20^ We noticed that ^13^C^16^O can be dismutated (similar to the Boudouard reaction) under an electron ionization source in mass spectrometry with a proportion of ^13^C^16^O^16^O generation. Therefore, we point out that it is essential to subtract a ^13^C^16^O^16^O baseline for all performance analyses. On the contrary, we observed merely trace amounts of ^13^C^18^O^18^O, which may be generated from direct ^18^O_2_ oxidation of homonuclear species ^13^C resulting from the dismutation reaction.

### Theoretical Calculations

Density functional theory (DFT) calculations were carried out using the Vienna Ab Initio Simulation Package (VASP).^34^ Our calculations used a plane-wave cutoff of 400 eV. We used the DFT+U correction^35^ with *U* – *J* = 3 eV. The PBE functional^36^ with Grimme’s D3 correction^37^ was used. The FeO_*x*_/Ir(111) slab was constructed with the experimental lattice constant for FeO. A 4 × 4 supercell with a 3 × 3 × 1 Monkhorst–Pack K-point mesh is used. The slab contains 3 layers of Ir, and a vacuum of 16 Å was placed above the surface. Dipole correction was also added perpendicular to the surface. The atomic positions in the FeO_*x*_ substrate layer and of the adsorbate are relaxed while the Ir layers are kept fixed in the calculations. The force convergence criteria for the geometry optimizations is 0.03 eV/Å. The FeO_*x*_ layer was initialized with an antiferromagnetic configuration in our calculations.

## Data Availability

The data that support the findings of this study are available in the Zenodo repository 10.5281/zenodo.7113212 and also from the corresponding author on request.

## Supporting Information Available

Sets of experimental STM images, line profiles, investigation of the CO/O_2_ TPD, and pulse-synchronized CO_2_ and water production, apparent activation energy, and extended calculated potential energy along a designed reaction pathway (PDF)

## References

[ref1] Hayek K.; Fuchs M.; Klötzer B.; Reichl W.; Rupprechter G. Studies of metal–support interactions with “real” and “inverted” model systems: reactions of CO and small hydrocarbons with hydrogen on noble metals in contact with oxides. Top. Catal. 2000, 13, 55–66. 10.1023/A:1009072519733.

[ref2] Rodriguez J. A.; Ma S.; Liu P.; Hrbek J.; Evans J.; Pérez M. Activity of CeOx and TiOx Nanoparticles Grown on Au(111) in the Water-Gas Shift Reaction. Science 2007, 318, 1757–1760. 10.1126/science.1150038. 18079397

[ref3] Ritter M.; Ranke W.; Weiss W. Growth and structure of ultrathin FeO films on Pt(111) studied by STM and LEED. Phys. Rev. B 1998, 57, 7240 10.1103/PhysRevB.57.7240.

[ref4] Vurens G. H.; Salmeron M.; Somorjai G. A. Structure, composition and chemisorption studies of thin ordered iron oxide films on platinum (111). Surf. Sci. 1988, 201, 129–144. 10.1016/0039-6028(88)90602-4.

[ref5] Zeuthen H.; et al. Structure of stoichiometric and oxygen-rich ultrathin FeO (111) films grown on Pd (111). J. Phys. Chem. C 2013, 117, 15155–15163. 10.1021/jp4042638.

[ref6] Ketteler G.; Ranke W. (2003). Heteroepitaxial growth and nucleation of iron oxide films on Ru (0001). J. Phys. Chem. B 2003, 107 (18), 4320–4333. 10.1021/jp027265f.

[ref7] Shaikhutdinov S. Strong Metal–support interaction and reactivity of ultrathin oxide films. Catal. Lett. 2018, 148, 2627–2635. 10.1007/s10562-018-2499-9.

[ref8] Fu Q.; et al. Interface-confined ferrous centers for catalytic oxidation. Science 2010, 328, 1141–1144. 10.1126/science.1188267. 20508127

[ref9] Kudernatsch W.; et al. Direct visualization of catalytically active sites at the FeO–Pt (111) interface. ACS Nano 2015, 9, 7804–7814. 10.1021/acsnano.5b02339. 26027877

[ref10] Pan Q.; Weng X.; Chen M.; et al. Enhanced CO oxidation on the oxide/metal interface: from ultra-high vacuum to near-atmospheric pressures. ChemCatChem. 2015, 7, 2620–2627. 10.1002/cctc.201500394.

[ref11] Zhang K.; Li L.; Shaikhutdinov S.; Freund H. J. Carbon Monoxide Oxidation on Metal-Supported Monolayer Oxide Films: Establishing Which Interface is Active. Angew. Chem., Int. Ed. 2018, 57, 1261–1265. 10.1002/anie.201710934. 29235223

[ref12] Saavedra J.; et al. The critical role of water at the gold-titania interface in catalytic CO oxidation. Science 2014, 345, 1599–1602. 10.1126/science.1256018. 25190716

[ref13] Ma Y.; Travaglia E.; Bana H.; Bignardi L.; Lacovig P.; Lizzit S.; Batzill M. Periodic modulation of graphene by a 2D-FeO/Ir (111) Moire interlayer. J. Phys. Chem. C 2017, 121, 2762–2770. 10.1021/acs.jpcc.6b11112.

[ref14] Sun Y. N.; Giordano L.; Goniakowski J.; Lewandowski M.; Qin Z. H.; Noguera C.; Shaikhutdinov S.; Pacchioni G.; Freund H. J. The interplay between structure and CO oxidation catalysis on metal-supported ultrathin oxide films. Angew. Chem., Int. Ed. 2010, 122, 4520–4523. 10.1002/ange.201000437. 20468018

[ref15] Merte L. R.; Bechstein R.; Peng G.; Rieboldt F.; Farberow C. A.; Zeuthen H.; Knudsen J.; Lægsgaard E.; Wendt S.; Mavrikakis M.; Besenbacher F. Water clustering on nanostructured iron oxide films. Nat. Commun. 2014, 5, 4193 10.1038/ncomms5193. 24979078

[ref16] Merte L. R.; et al. Water-mediated proton hopping on an iron oxide surface. Science 2012, 336, 889–893. 10.1126/science.1219468. 22605771

[ref17] Meier M.; Hulva J.; Jakub Z.; Pavelec J.; Setvin M.; Bliem R.; Schmid M.; Diebold U.; Franchini C.; Parkinson G. S. Water agglomerates on Fe_3_O_4_ (001). Proc. Natl. Acad. Sci. U.S.A. 2018, 115, 5642–5650. 10.1073/pnas.1801661115. PMC6016784 29866854

[ref18] Gamba O.; Hulva J.; Pavelec J.; Bliem R.; Schmid M.; Diebold U.; Parkinson G. S. The role of surface defects in the adsorption of methanol on Fe_3_O_4_ (001). Top. Catal. 2017, 60, 420–430. 10.1007/s11244-016-0713-9. 32025174 PMC6979651

[ref19] Bonanni S.; Aït-Mansour K.; Hugentobler M.; Brune H.; Harbich W. An experimental setup combining a highly sensitive detector for reaction products with a mass-selected cluster source and a low-temperature STM for advanced nanocatalysis measurements. Eur. Phys. J. D 2011, 63, 241–249. 10.1140/epjd/e2011-10532-7.

[ref20] de Groot J. G. An Experimental Setup for Heterogeneous Catalysis on Atomically Defined Metal Nanostructures. Ph.D. Thesis, École Polytechnique Fédérale de Lausanne, Switzerland, 2021.

[ref21] Zhang K.; Li L.; Goniakowski J.; Noguera C.; Freund H. J.; Shaikhutdinov S. Size effect in two-dimensional oxide-on-metal catalysts of CO oxidation and its connection to oxygen bonding: An experimental and theoretical approach. J. Catal. 2021, 393, 100–106. 10.1016/j.jcat.2020.11.022.

[ref22] Liu L.; Zhou F.; Wang L.; Qi X.; Shi F.; Deng Y. Low-temperature CO oxidation over supported Pt, Pd catalysts: Particular role of FeOx support for oxygen supply during reactions. J. Catal. 2010, 274, 1–10. 10.1016/j.jcat.2010.05.022.

[ref23] Liu Y.; Zhang R.; Lin L.; Wang Y.; Liu C.; Mu R.; Fu Q. Direct observation of accelerating hydrogen spillover via surface-lattice-confinement effect. Nat. Commun. 2023, 14, 613 10.1038/s41467-023-36044-8. 36739275 PMC9899253

[ref24] Kraushofer F.; et al. Self-limited growth of an oxyhydroxide phase at the Fe_3_O_4_ (001) surface in liquid and ambient pressure water. J. Chem. Phys. 2019, 151, 154702 10.1063/1.5116652. 31640372

[ref25] Hagedorn C. J.; Weiss M. J.; Weinberg W. H. Dissociative chemisorption of hydrogen on Ir (111): Evidence for terminal site adsorption. Phys. Rev. B 1999, 60, 14016 10.1103/PhysRevB.60.R14016.

[ref26] Karim W.; Spreafico C.; Kleibert A.; et al. Catalyst support effects on hydrogen spillover. Nature 2017, 541, 68–71. 10.1038/nature20782. 28054605

[ref27] Conner W. C.; Falconer J. L. Spillover in heterogeneous catalysis. Chem. Rev. 1995, 95, 759 10.1021/cr00035a014.

[ref28] Doudin N.; et al. Understanding heterolytic H2 cleavage and water-assisted hydrogen spillover on Fe3O4 (001)-supported single palladium atoms. ACS Catal. 2019, 9, 7876–7887. 10.1021/acscatal.9b01425.

[ref29] Digne M.; Sautet P.; Raybaud P.; Euzen P.; Toulhoat H. Use of DFT to achieve a rational understanding of acid–basic properties of γ-alumina surfaces. J. Catal. 2004, 226, 54–68. 10.1016/j.jcat.2004.04.020.

[ref30] Saavedra J.; Whittaker T.; Chen Z.; Pursell C. J.; Rioux R. M.; Chandler B. D. Controlling activity and selectivity using water in the Au-catalysed preferential oxidation of CO in H2. Nat. Chem. 2016, 8, 584–589. 10.1038/nchem.2494. 27219703

[ref31] Bollinger M. A.; Vannice M. A. A kinetic and DRIFTS study of low-temperature carbon monoxide oxidation over Au–TiO_2_ catalysts. Appl. Catal. B: Environ. 1996, 8, 417–443. 10.1016/0926-3373(96)90129-0.

[ref32] Debeila M. A.; Wells R. P. K.; Anderson J. A. Influence of water and pretreatment conditions on CO oxidation over Au/TiO2–In2O3 catalysts. J. Catal. 2006, 239, 162–172. 10.1016/j.jcat.2006.01.028.

[ref33] Brune H.; Röder H.; Bromann K.; Kern K. Kinetic processes in metal epitaxy for Ag/Pt(111) studied with variable temperature STM. Thin Solid Films 1995, 264, 230 10.1016/0040-6090(94)05821-0.

[ref34] Kresse G.; Furthmuller J. Efficiency of ab-initio total energy calculations for metals and semiconductors using a plane-wave basis set. J. Comput. Mater. Sci. 1996, 6, 15 10.1016/0927-0256(96)00008-0. 9984901

[ref35] Dudarev S. L.; Botton G. A.; Savrasov S. Y.; Humphreys C. J.; Sutton A. P. Electron-energy-loss spectra and the structural stability of nickel oxide: An LSDA+U study. Phys. Rev. B 1998, 57, 1505 10.1103/PhysRevB.57.1505.

[ref36] Perdew J. P.; Burke K.; Ernzerhof M. Generalized Gradient Approximation Made Simple. Phys. Rev. Lett. 1997, 78, 1396 10.1103/PhysRevLett.78.1396. 10062328

[ref37] Grimme S.; Antony J.; Ehrlich S.; Krieg S. A consistent and accurate ab initio parametrization of density functional dispersion correction (DFT-D) for the 94 elements H-Pu. J. Chem. Phys. 2010, 132, 154104 10.1063/1.3382344. 20423165

