# Supplementary material for: Probing Catalytic Sites and Adsorbate Spillover on Ultrathin FeO2–x Film on Ir(111) during CO Oxidation
Source: ACS Nano. 2024 Feb 20;18(9):7114–22. doi: 10.1021/acsnano.3c11400 (PMC10919091; doi:10.1021/acsnano.3c11400)
Supplement: Supplementary file 1 — nn3c11400_si_001.pdf [file nn3c11400_si_001.pdf]

Probing catalytic sites and adsorbate spillover on ultrathin FeO_{2-x} film on Ir(111) during CO Oxidation

Hao Yin[1*], Yu-Wei Yan[2], Wei Fang[2], Harald Brune[1*]

1. Institute of Physics, École Polytechnique Fédérale de Lausanne (EPFL), 1015 Lausanne, Switzerland
2. Department of Chemistry, Collaborative Innovation Center of Chemistry for Energy Materials, Shanghai Key Laboratory of Molecular Catalysis and Innovative Materials, Fudan University, Shanghai 200438, China

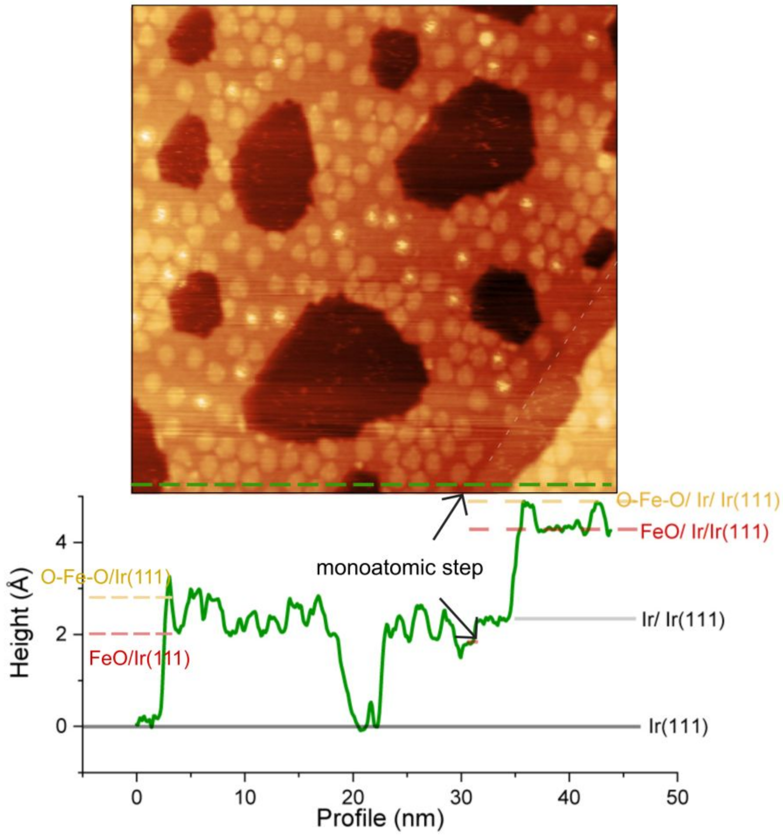

Fig. S1. STM image of a 0.6 ML FeO_{2-x} on Ir(111) with height profile (green line), 45 nm* 45 nm.

Fig. S1 shows the STM image of a partially oxidized FeO_{2-x} film on Ir(111). The coverage of the oxide film is nearly 0.6 ML, there is a monoatomic step in the lower right corner (marked with a white dash) The O-Fe-O structure appears as round and bright patches, whereas the 10-20 nm diameter pits refer to uncovered Ir surface. *Insufficient*‘ O_2 treatment results that trilayer O-Fe-O structures co-exist with pristine bilayer FeO depending on the oxygen chemical potential. Under the same tunneling condition, we measure a sharp Ir monoatomic step with ~2.3 Å height modulation, close to the ideal interlayer distance (221.7 pm). A profile line on O-rich film reveals height modulation of about 0.5 Å in amplitude, which is slightly shorter than the one on FeO_2/Pt(111)[3] (0.6 Å) due to the different d band center of Ir and Pt.

`

Fig. S2. O_2 (36 AMU) Temperature-programmed spectrum recorded on FeO_{2-x}/ Ir(111). The temperature ramp is 350 K to 820 K with a 3 K/min heating speed.

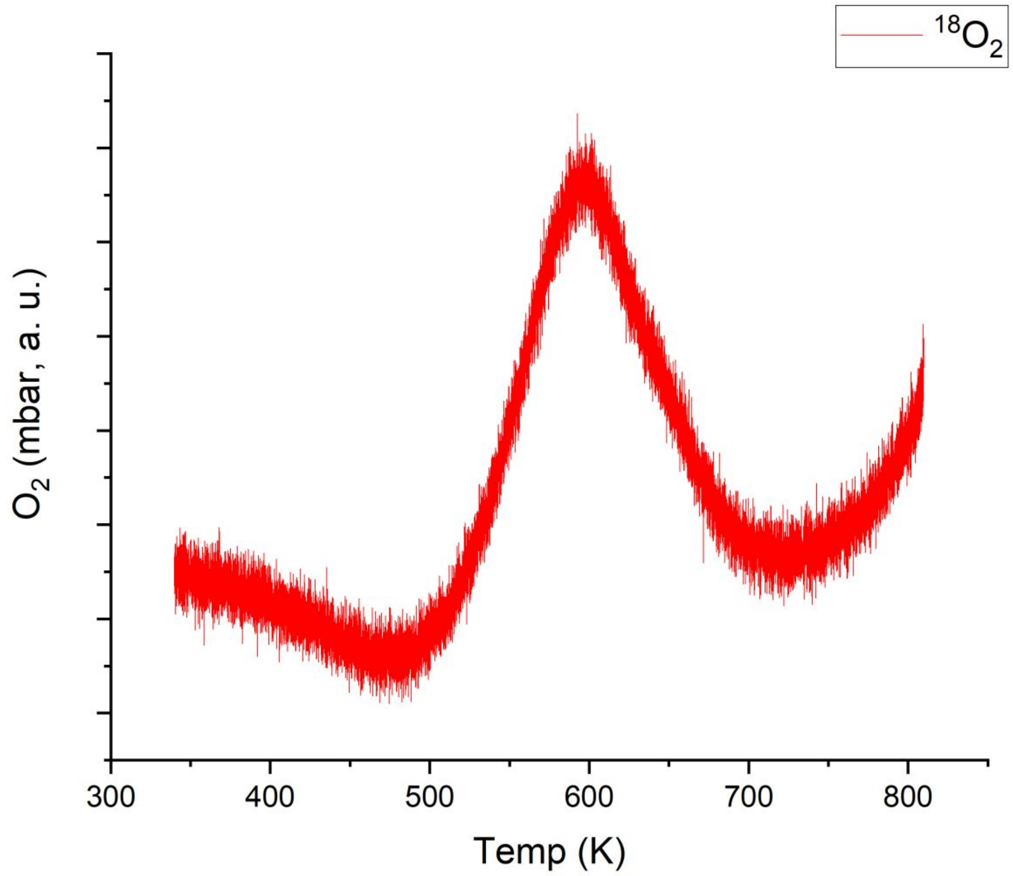

We observed a desorption peak at 600 K denoting the weakly bonded oxygen layer, which is lower than other reports[2] (peak at 700-800 K). This is mainly due to the lower heating speed (3 K/min) we applied compared with others[2] (120 K/min). Pristine FeO films decompose at temperatures as high as 1170 K[3].

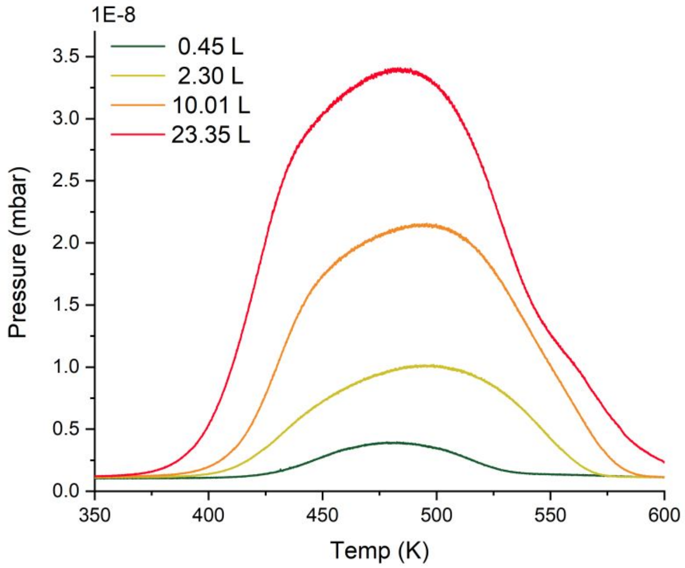

Fig. S3. CO-TPD experiment on Ir(111) with different amounts of CO dose at 300 K. The heating rate is 0.5 K/s.

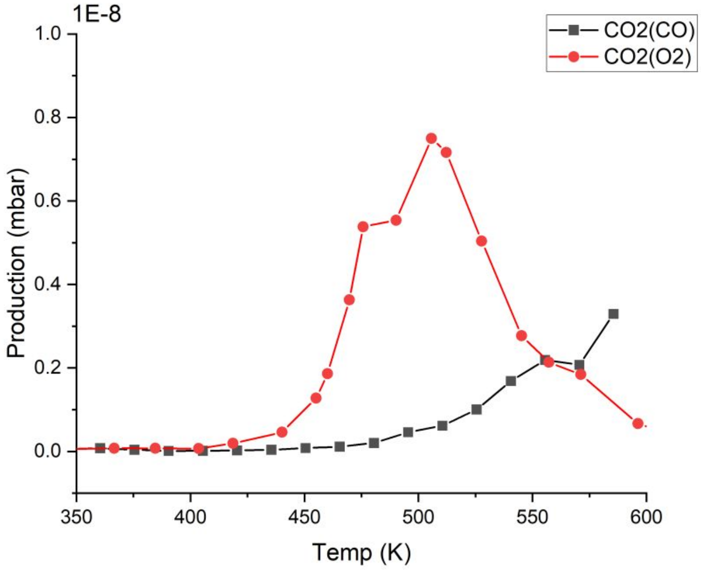

Fig. S4. Pulse-synchronized CO_2 production from Ir(111) surface.

CO molecules start to desorb from Ir(111) surface at 400 K and the desorption peak appears at ca. 500 K. The interaction between CO and Ir surface determines the CO_2 performance with three significant stages: 1, Below 400 K, the CO_2 generation is prohibited due to strong CO adsorption, also known as poison effects; 2, between 400-550K, there are still active sites for O_2 adsorption on CO-dominant surface where CO_2 generation is more sensitive to O_2 pulse; 3, above 550 K, CO-dominant surface turn to O-dominate surface where CO_2 generation

is more sensitive to CO pulse. In order to minimize the interference of CO_2(CO) generation from the Ir surface, we choose 500 K as the reaction temperature on FeO_x/Ir.

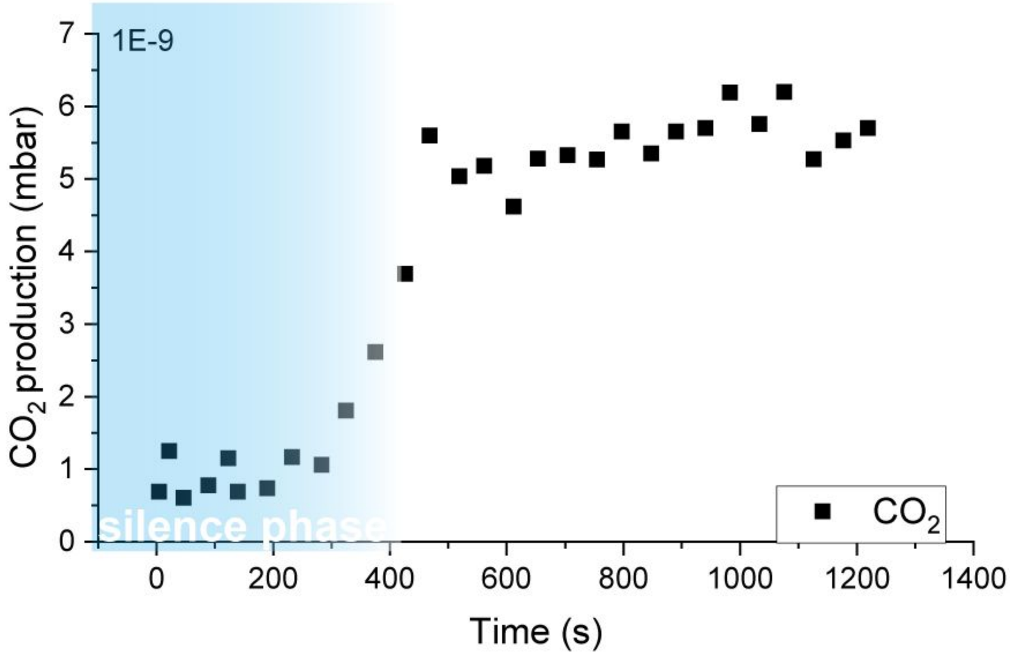

Fig. S5. CO_2(CO) performance on FeO_{2-x}/ Ir(111) with repeatedly CO/O_2 pulse with second-level intervals.

As shown in Fig. 1a, for FeO_{2-x}/ Ir, the distribution of trilayer WBOs as active phases on the surface is relatively inhomogeneous. The interfacial fast reactive region is gradually resumed during the O_2 dose period, as seen in Fig. S5 0-400 s period (blue shade). Once the interfacial WBOs fully recovered, the CO_2(CO) generation reaches a fast plateau: a cycle of interfacial WBOs consumption and recovery. Before that, WBOs on terraces contributed to CO_2 generation with a lower reaction rate, which we regard as the *silence phase* or *low-reactivity phase*.

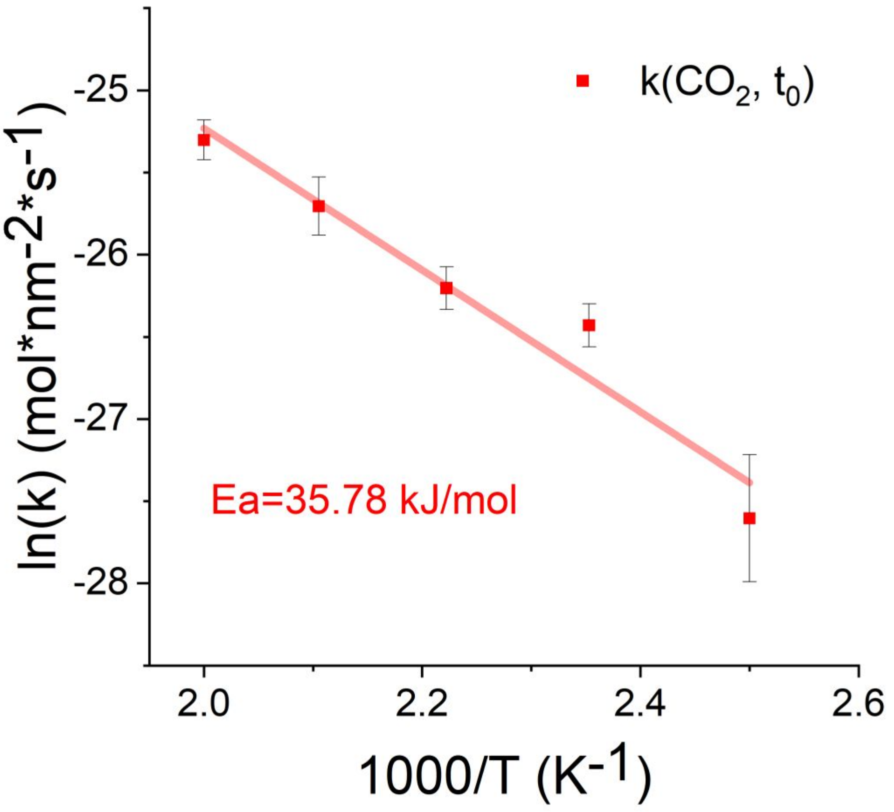

Fig. S6. Apparent activation energy of CO oxidation on O-Fe-O / Ir(111) interline.

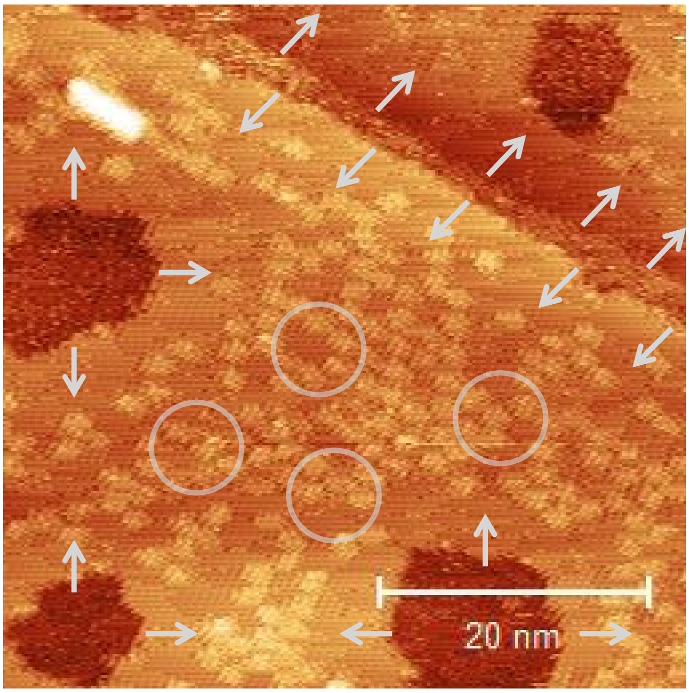

Fig. S7. STM images of 0.6 ML FeOx/Ir in Fig. 3B after another 30 min CO treatment (60 mins CO treatment in total) at 500 K. Arrow indication the the reaction initially begin at FeO2/Ir boundary, and cycle indicate the reaction start at the FeO_2 terrance.

Compared with the surface morphology in Fig. 3B (30 min CO treatment), the coverage of the trilayer oxygen moiré pattern further decreases after another 30 min CO treatment not only at the FeO_x/Ir interface but also at the FeO terrace. Consistent with the CO_2 performance, we confidently identify that both weakly bonded oxygen at the interface and terrace are active phases for CO oxidation. We notice that similar to images in Fig. 3B, even though the WBOs at the interface are fully consumed, there are still a large amount of WBOs remaining at the terrace, indicating that the WBOs at the terrace possess a lower reactivity than one at the interface.

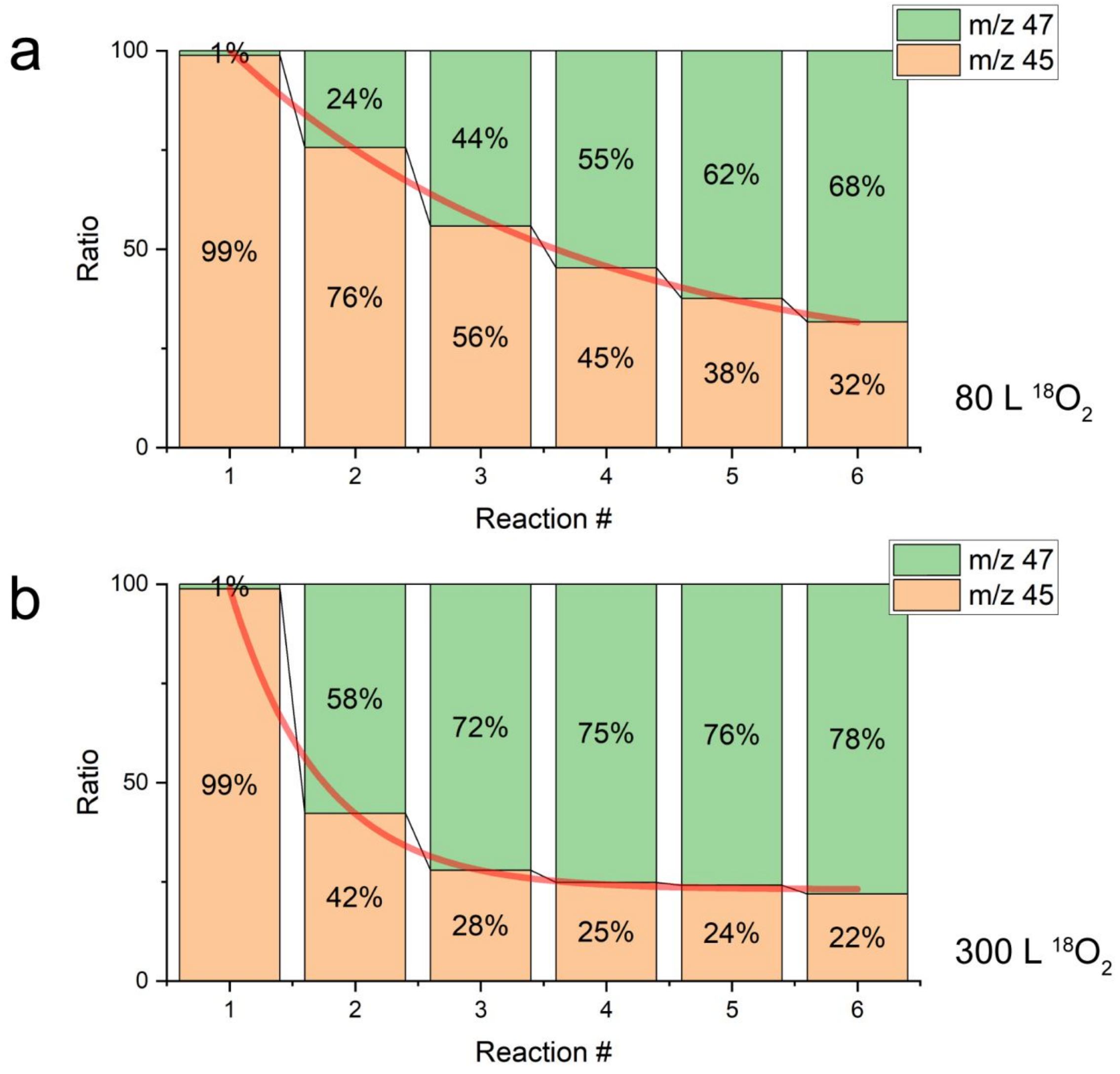

Fig. S8. The initial CO_2 isotopic ratio ($^{13}C^{16}O^{18}O$: green, $^{13}C^{16}O^{16}O$: brown) develops with reaction cycles where FeO and CO are the sources of ^{16}O and O_2 is the source of ^{18}O with different amounts of O_2 during the re-oxidation period a) ~80 L and b) ~300 L.

As mentioned, due to the exchange between dissociated ^{18}O atom and the remaining ^{16}O atom on oxide during oxygen treatment at 570 K, the $^{13}C^{16}O^{18}O$ products gradually dominate compared with $^{13}C^{16}O^{16}O$. Furthermore, when the amount of oxygen increase, the ratio changes faster. As shown in Fig, S3 and S4, the CO molecules start to desorb from the Ir surface with remaining sites for oxygen dissociative adsorption. Based on classical collision theory, with increasing oxygen molecules, the frequency of collisions on the surface consequently turns faster as well as the dissociation rate. The directly positive (and nearly linear) correlation between oxygen pressure and ratio dynamics indicates that the rate-limiting step for WBO re-generation is the dissociation of oxygen on the surface rather than surface O atoms reaction with interfacial Fe-O.

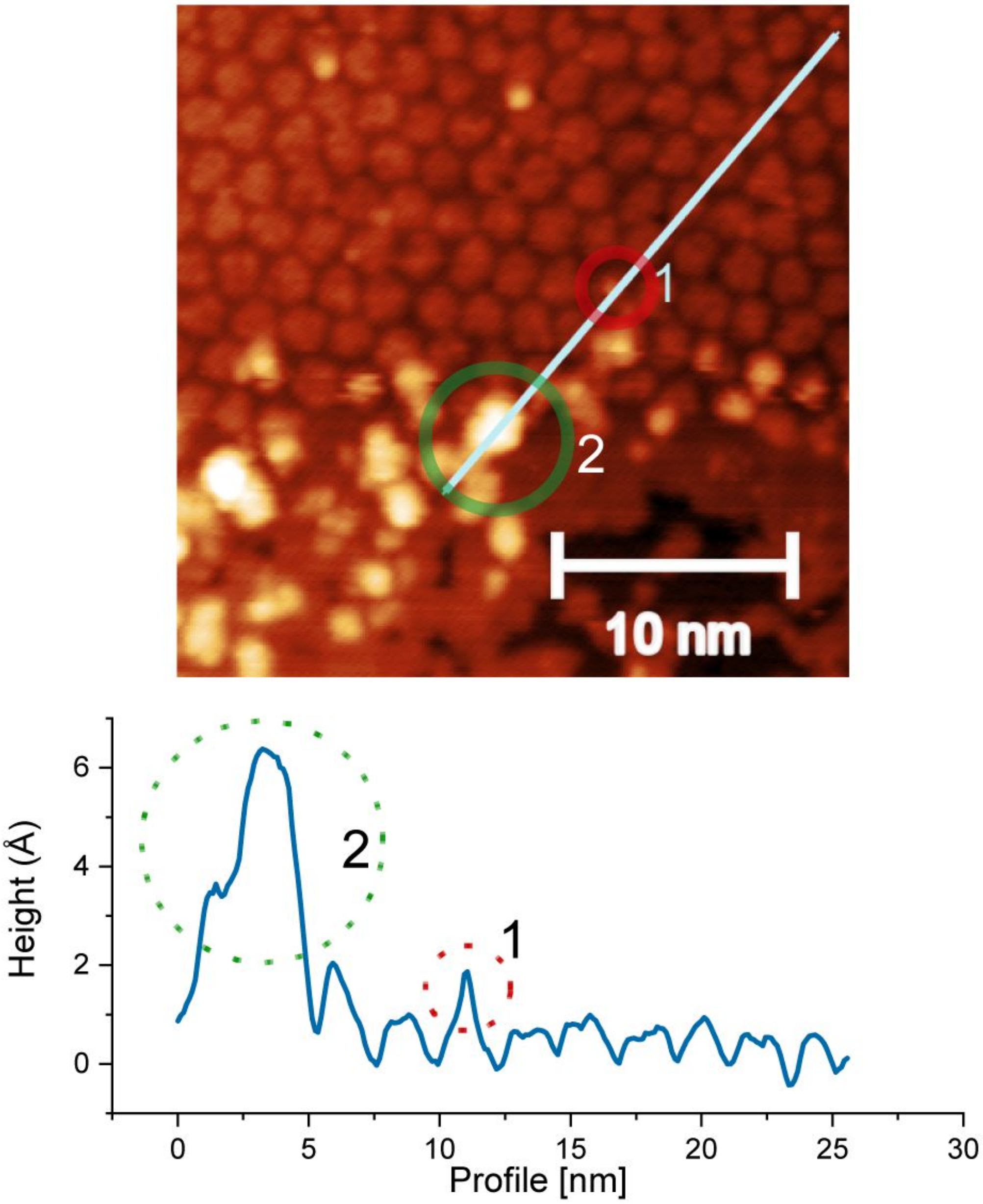

Fig. S9. STM image (2.0 V, 0.1 nA) of the FeO_2 film on Ir(111) with the existence of surface hydroxyl species and water clusters, acquired at 300 K after ca. 100 L hydrogen treatment at 500 K.

After hydrogen treatment, we observe larger protrusions on the surface, especially at the FeO_{2-x}/Ir interface. According to the height profile, there are mainly two different types of protrusions: one is small (ca. 1 Å, #1 in Fig. S9) and not only appears at the interface but also at the terrace, the other is much larger (ca. 2.5 Å for monolayer and ca. 5 Å for bilayer) and mostly located at the interface. According to reference[6,7], we regard the former as surface OH species and the latter as water clusters which may form a framework due to hydrogen bonds. Limited by the current spatial resolution, it is a challenge for us to resolve their fine structure.

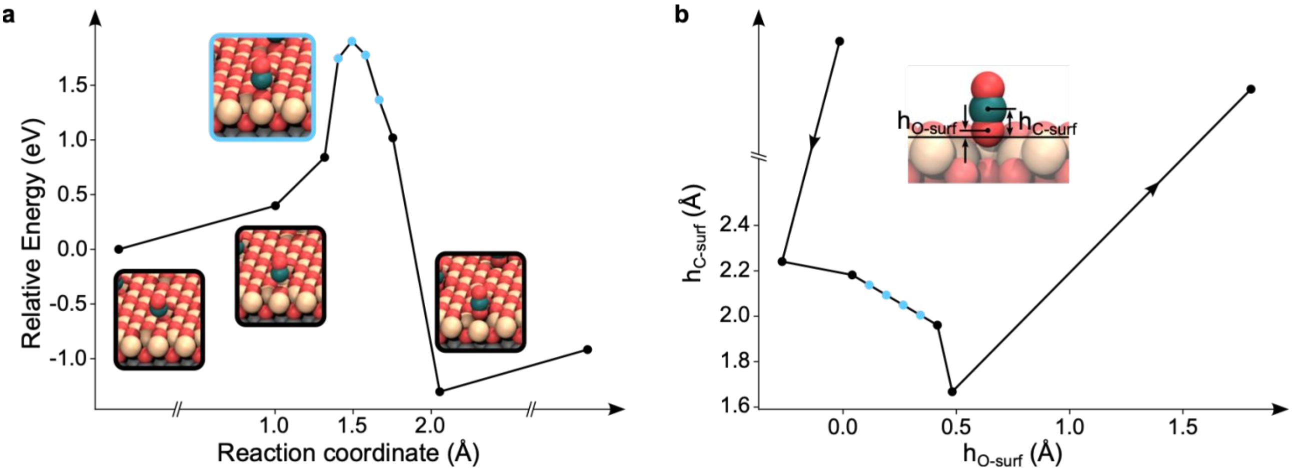

Fig. S10. (a) Potential energy along a designed reaction pathway for CO reaction with a WBO on FeO_2 terrace, starting from physical adsorption and ending with the state of free carbon dioxide with the slab without an O atom. The insets show the geometries along the reaction path. **(b)** The reaction pathway is defined using two reaction coordinates: the distance between C and the surface O (RCO) and the height of the CO molecule (hCO). The black points mean that the geometry is relaxed with the two reaction coordinates fixed, while the blue points represent linear interpolation between the geometries represented by two black points.

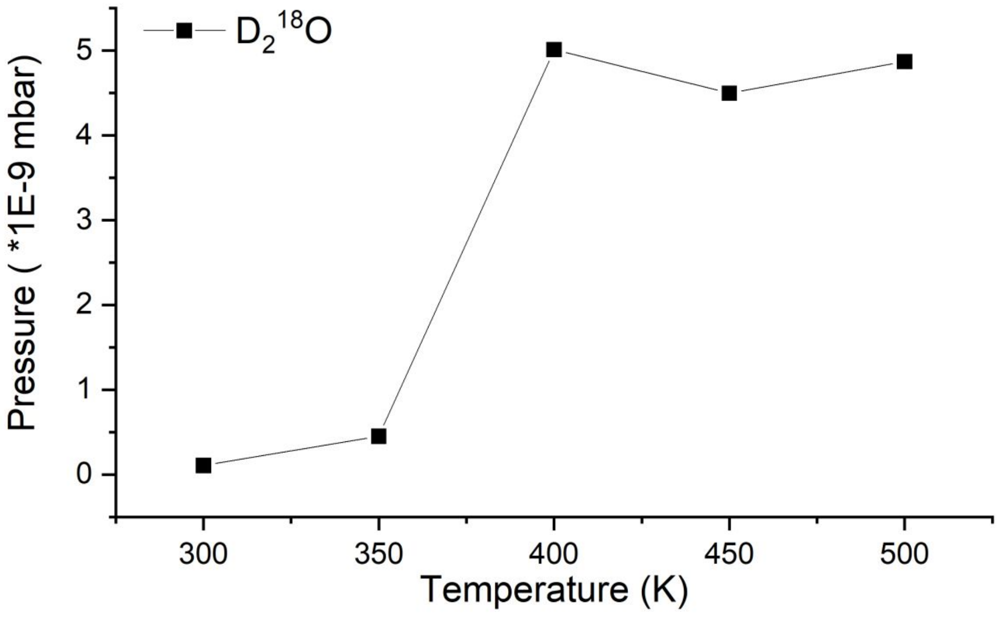

Fig. S11. Water generation (m/z=22) on $Fe^{18}O_2$/Ir after D_2 treatment at different temperatures.

Here we dosed D_2 on fresh-oxidized FeO_2/Ir at five temperature set points. A strong $D_2{}^{18}O$ production was observed above 400 K resulting from the dissociation of surface hydroxyl species and desorption of surface water cluster. The results match with former STM images and can be rationalized with the hydrogen spillover mechanism as shown in Fig. 6.

Reference

1. Zeuthen, H., Kudernatsch, W., Merte, L. R., Ono, L. K., Lammich, L., Besenbacher, F. & Wendt, S. Unraveling the edge structures of platinum (111)-supported ultrathin FeO islands: the influence of oxidation state. *ACS Nano* **9**, 1, 573–583. (2015).
2. Zhang, K., Li, L., Goniakowski, J., Noguera, C., Freund, H. J. & Shaikhutdinov, S. Size effect in two-dimensional oxide-on-metal catalysts of CO oxidation and its connection to oxygen bonding: An experimental and theoretical approach. *Journal of Catalysis*, **393**, 100-106 (2021).
3. Sun, Y. N., Giordano, L., Goniakowski, J., Lewandowski, M., Qin, Z. H., Noguera, C., Shaikhutdinov, S., Pacchioni, G. & Freund, H. J. The interplay between structure and CO oxidation catalysis on metal-supported ultrathin oxide films. *Angew. Chem. Int. Ed.* **122**, 4520-4523 (2010).
4. Merte, L. R., Bechstein, R., Peng, G., Rieboldt, F., Farberow, C. A., Zeuthen, H., Knudsen, J., Lægsgaard, E., Wendt, S., Mavrikakis, M & Besenbacher, F. Water clustering on nanostructured iron oxide films. *Nat Commun* **5,** 4193 (2014).
5. Meier, M., Hulva, J., Jakub, Z., et al. Water agglomerates on Fe3O4 (001). *Proceedings of the National Academy of Sciences*, **115**, 5642-5650 (2018).
